# Progress in Medicine

**Published:** 1900-06-16

**Authors:** 


### Progress in Medicine.

DIABETES.

Since nitro-propiol is reduced by glucose so as to form indigo, it has been lately recommended as a test for sugar in the urine.[1] It is said to indicate as little as ·05 per cent of grape sugar, and not to be affected by kreatinin, glycuronic acid, salicylic acid, or iodine. Tablets have been manufactured to simplify its use, and are known as Teusch's nitro-propiol test. B. Gordon[2] claims that there is only one body, aldehyde, which interferes with its reaction, and that the percentage of sugar which can be discovered is less than that possible under the fermentation test. He carries it out by boiling the propiol for 30 seconds in a test tube with a dilute solution containing glucose, when a deep blue colour appears. Phenyl hydrazin as a test for sugar has been made the subject of experiments by Coriat,[3] who finds that each variety of sugar forms a definite special type of crystal with it, and that it is possible to distinguish any of the sugars by their crystalline form alone. For clinical use the phenyl hydrazin should be in excess, but it is not necessary to remove albumen beforehand. One reason for its importance is that few, if any, of the substances which interfere with Fehling's or Nylander's reagents interfere with the accuracy of this test. As a quick and perfectly reliable plan R. Saundby[4] mentions his method of filtering the urine through animal charcoal seven or eight times before applying Fehling's reagent. This can be done in 10 minutes without any difficulty, and, as the filtration removes all the reducing substances except sugar, we have a very perfect and simple test. The fermentation reaction, valuable as it is, may be vitiated by traces of sugar in the yeast, and, moreover, takes a considerable time. Hence he employs the combined filtration and Fehling's methods in all doubtful cases in his own practice.

**Non-diabetic Glycosuria.**—He has met with 69 cases of non-diabetic urine which reduced copper, and in 11 of these the reduction was not due to sugar. Such glycosuria may be temporary, and caused by food, since even healthy persons excrete sugar if they consume at one time 300 to 500 grains of grape sugar. Other cases are due to digestive disturbances, hepatitis, alcoholism, gout, renal and biliary calculi; and others to nerve diseases, infectious disorders, senile decay, and lactation. The alcoholic are a numerous class, and both in them and in the hepatic and gastric cases the glycosuria probably arises from sudden discharges of sugar from the liver in quantities greater than can be used up in the body. In any non-diabetic case a rigid diet is undesirable, especially in the senile form, and though the author sees no objection to cutting off sugar, he would not stop the bread or potatos. The physiological glycosuria which is found in pregnancy and lactation should not cause any grave prognosis, or lead us to insist on any special diet. H. Strauss[5] has recently studied the glycosuria which occurs after injuries and shock. In 109 cases of traumatic neurosis he found that 30·3 per cent. showed glycosuria. Töpfer[6] has shown that diabetes in rabbits may be caused by a sort of infection, for he has succeeded in producing it by injecting under the skin the contents of the intestines of a diabetic patient. Other rabbits which had been injected with fluid from the bowels of healthy persons remained unaffected. Similar results were obtained by injecting dogs. Hammerschlag, in continuation of this work, claims to have isolated from the intestinal contents of diabetics a bacillus, cultures of which, when injected under the skin, produced a marked and lasting glycosuria. Pary[7] has demonstrated the production of glycosuria by phlorizin in a kidney which had been removed from the body. Provided the circulation was kept up sugar was regularly formed from a proteid molecule, and through the action of the drug it was enabled to pass through the renal epithelium. The pathology of diabetic coma may be mentioned here with respect to certain investigations of K. Grube. He was led to think that Beta-amido-butyric acid is a probable cause of this condition, and by administering the acid to cats hypodermically he succeeded (1) in producing coma; (2) in causing respiration of a type peculiar to this coma; (3) while in the urine diacetic acid and acetone could be detected. Therefore he believes that this is one at least of the substances which produce diabetic coma.

The use of uranium nitrate in controlling glycosuria has been revived by S. West. Hubert Bond[8] also gave it to nine patients with considerable success. In one case the sugar disappeared entirely and did not return. The patient had been a sufferer for eight or ten years, and was treated with doses varying from three to ten grains three times a day for 12 months, but he was not placed on a strict diet. In another case the dose was gradually increased up to 30 grains three times a day, which was continued for 12 months. As a rule, the patient received at first three grains of the drug twice a day after food, in an ounce or more of water to avoid gastric irritation. No ill effects were noticed in any of them.

Another method of treatment advocated by Kolisch[9] consists in feeding the patients entirely on a vegetable diet, as more alkaline, less toxic, and less irritating than any other. The starches and sugars are more or less excluded, but green vegetables, eggs, fatty matters, fruits, nuts, and mushrooms, with tea and coffee, are given freely. In order to obtain thoroughly alkaline urine, Kolisch administers three ounces of bicarbonate of soda daily to patients who are taking this diet. A fatal case in a child of five years is recorded by Leonard Lees and Milligan,[10] which is remarkable for its very rapid course. Eleven days before death he was treated for constipation, but otherwise appeared to be in good health. Five days later vomiting and thirst came on. Then wasting, pallor, sighing respiration, and restlessness continued till a fatal state of coma ended the scene. The day before death large quantities of both sugar and albumen were found in the urine, and the peculiar sweet odour which is so well known was recognised in his breath.

**Surgery in Diabetic Patients.**—A. L. Fisk[11] has reviewed the question of the possibility of surgical treatment when diabetes is present, and considers that, though such patients are not good subjects, operations may be properly undertaken if complete asepsis be maintained and proper cases are chosen. Thus, in many instances, the sugar is actually due to the surgical lesion, such as a blow on the head, or an infection by staphylococcus aureus, and the glycosuria here does not seem to render the operation any more dangerous. Generally speaking, the author urges that the greatest judgment must be used in the selection of cases, the blood supply of the tissues should not be interfered with more than necessary, and every possible care should be taken to avoid sepsis from without. E. Külz, at his death, left behind him observations on a vast number of diabetics, and his fellow-worker, Rumpf, has published the analyses of nearly 700 of these clinical histories. According to their view, there is but one kind of diabetes, which appears under various forms as the resistance of the individual differs. The treatment in the main consisted of careful dietetic management adapted to each individual from day to day, so as to increase his power of assimilating starch. The effects of each change on the sugar, acetone, urea, &c., are carefully recorded, and the volume forms, perhaps, the most elaborate collection of facts on the subject of diabetes ever published.[12] Bronze or pigmented diabetes is discussed by E. Berg,[13] who reports an instance of this extremely rare complaint following after an attack of exophthalmic goitre. The diabetes was of a severe and progressive type, the face, arms, and thighs were markedly pigmented, and the liver affected with hypertrophic cirrhosis, which caused enlargement of the superficial abdominal veins with œdema of the legs and ascites. The pigment in this disease has been shown to be due to an iron compound, and the author regards this as removed from the blood by the dilute sugar-containing serum. The liver cirrhosis is, he holds, a secondary phenomenon, and due to the deposit of pigment in a liver suffering from the ordinary congestion of diabetes.

Meltzer, in commenting on this case, remarks that bronzed diabetes has never been hitherto observed in a woman, and he would prefer to consider it as an instance of pigmentation due to the goitre, which had been complicated by ordinary diabetes. According to another theory of bronze diabetes, the first stage consists in some break-up of the blood cells, which leads to interstitial cirrhosis from the irritation of the liver by the deposited pigment. In a similar manner interstitial pancreatitis is produced. Seventeen out of 24 recorded cases of bronze diabetes presented this complication, and it is possible that it existed also in the others. Thus it is the pancreatitis which, on this theory, is the cause of the glycosuria, and the latter generally appears at a late stage of the disease.

[1] Lancet, Feb. 17. [2] Lancet, May 5. [3] New York Med., Dec. 9. [4] Brit. Med. Jour., April 14. [5] Lancet, Jan. 20. [6] La Semaine Med., Dec. 10. [7] Lancet, Mar. 10. [8] Practitioner, p. 257-99. [9] La Semaine Med., Jan. 24. [10] Lancet, Dec. 23. [11] Annals of Surgery, April. [12] Klinische Erfahrungen ü Diabetes, E. Külz and Rumpf. [13] Med. Rec., Dec. 16.

